# Prion protein inhibits fast axonal transport through a mechanism involving casein kinase 2

**DOI:** 10.1371/journal.pone.0188340

**Published:** 2017-12-20

**Authors:** Emiliano Zamponi, Fiamma Buratti, Gabriel Cataldi, Hector Hugo Caicedo, Yuyu Song, Lisa M. Jungbauer, Mary J. LaDu, Mariano Bisbal, Alfredo Lorenzo, Jiyan Ma, Pablo R. Helguera, Gerardo A. Morfini, Scott T. Brady, Gustavo F. Pigino

**Affiliations:** 1 Laboratorio de Neuropatología Experimental, Instituto de Investigación Médica Mercedes y Martín Ferreyra, INIMEC-CONICET-Universidad Nacional de Córdoba, Córdoba, Argentina; 2 Department of Anatomy and Cell Biology, University of Illinois at Chicago, Chicago Illinois, United States of America; 3 Marine Biological Laboratory, Woods Hole, Massachusetts, United States of America; 4 Harvard Program in Therapeutic Science, Harvard Medical School, Boston, Massachusetts, United States of America; 5 Laboratorio de Neurobiología Experimental, Instituto de Investigación Médica Mercedes y Martín Ferreyra, INIMEC-CONICET-Universidad Nacional de Córdoba, Córdoba, Argentina; 6 Center for Neurodegenerative Science, Van Andel Research Institute, Grand Rapids, Michigan, United States of America; IRCCS—Mario Negri Institute for Pharmacological Research, ITALY

## Abstract

Prion diseases include a number of progressive neuropathies involving conformational changes in cellular prion protein (PrP^c^) that may be fatal sporadic, familial or infectious. Pathological evidence indicated that neurons affected in prion diseases follow a dying-back pattern of degeneration. However, specific cellular processes affected by PrP^c^ that explain such a pattern have not yet been identified. Results from cell biological and pharmacological experiments in isolated squid axoplasm and primary cultured neurons reveal inhibition of fast axonal transport (FAT) as a novel toxic effect elicited by PrP^c^. Pharmacological, biochemical and cell biological experiments further indicate this toxic effect involves casein kinase 2 (CK2) activation, providing a molecular basis for the toxic effect of PrP^c^ on FAT. CK2 was found to phosphorylate and inhibit light chain subunits of the major motor protein conventional kinesin. Collectively, these findings suggest CK2 as a novel therapeutic target to prevent the gradual loss of neuronal connectivity that characterizes prion diseases.

## Introduction

Prion diseases include a number of fatal sporadic, familial and infectious neuropathies affecting humans and other mammals [1]. As observed in most adult-onset neurodegenerative diseases [2], neurons affected in prion diseases follow a dying back pattern of degeneration, where synaptic dysfunction and loss of neuritic connectivity represent early pathogenic events that long precede cell death [3, 4]. Toxic effects of prion protein (PrP) have been shown in various cellular and animal models [5–7]. An intriguing characteristic of prion diseases is the nature of prion, a pathogen devoid of nucleic acid [8]. The infectious form of prion disease involves a conformation-related conversion of the cellular form of PrP (PrP^c^) to a mildly protease-resistant aggregated, and self-propagating species termed PrP scrapie (PrP^Sc^) [1, 9]. However, genetic and experimental evidence suggest that additional factors affecting PrP conformation may similarly promote neuronal pathology. For example, mutant PrP-related familial forms of prion diseases have been identified which do not involve the PrP^Sc^ conformation [1, 10]. In addition, aggregated, non-infectious oligomeric PrP has also been shown to induce neurotoxicity [4, 9, 11, 12]. Further, results from our prior work indicate that intracellular accumulation of full-length PrP^c^ (PrP-FL) alone suffices to induce progressive neuronal toxicity in cultured neurons and severe ataxia in mice [5, 13–16]. Collectively, these observations suggest that a variety of factors, including increased PrP^c^ dosage and conformation-dependent conversion of PrP^c^ to various neurotoxic species may underlie prion disease pathology, thus providing a common framework for seemingly diverse prion disease variants.

The dying-back pattern of degeneration observed in neurons affected in prion diseases strongly suggests that pathogenic forms of PrP may interfere with cellular processes relevant to the maintenance of neuronal connectivity, such as fast axonal transport (FAT). The unique dependence of neuronal cells on FAT has been documented by genetic findings that link loss of function mutations in molecular motors to dying back degeneration of selected neuronal populations [17–23]. Significantly, microscopic analysis documented deficits in anterograde and retrograde FAT in PrP^sc^-inoculated mice concurrent with the development of prion disease symptoms [24, 25]. However, whether pathogenic PrP^c^ *directly* affects FAT has not yet been evaluated, and mechanisms underlying the FAT deficits observed in prion diseases remain largely unknown.

A large body of experimental evidence indicates that various misfolded neuropathological proteins compromise FAT by promoting alterations in the activity of protein kinases involved in the regulation of microtubule-based motor proteins [26–28]. Consistent with findings in a variety of adult-onset neurodegenerative diseases, aberrant patterns of protein phosphorylation represent a well-established hallmark of prion diseases. Further, several kinases known to affect FAT are reportedly deregulated in prion diseases, including GSK3 [29], PI3K [30], JNK [31], and casein kinase 2 (CK2) [32, 33], Based on these precedents, we set out to determine whether PrP-FL inhibits FAT directly and, if so, determine whether specific protein kinases mediate such effects.

## Materials and methods

### Cell culture

Hippocampal neuronal cultures were prepared from wild type B6SJL mouse embryos at day 16 of gestational age [34]. After dissection, the cortical or hippocampal tissue was incubated in 0.25% trypsin in Hank’s for 16 min at 37°C, followed by dissociation and plating of the cell suspension in culture dishes or glass coverslips covered with poly-D-lysine (0.5 mg/ml), at a density of 53 cells/cm^2^ for immunocytochemistry or 350 to 1050 cell/cm^2^ for biochemical analysis. The cultures were plated in DMEM plus 10% iron-supplemented calf serum (HyClone, Logan, UT) for 2 hours, and then replaced with Neurobasal media supplemented with B27 (Life Technologies, Grand Island, NY).

Animals were housed in the University of Illinois at Chicago Biological Resource Laboratory. All animal work was done according to guidelines established by the NIH and are covered by appropriate institutional animal care and use committee protocols from the University of Illinois at Chicago Animal Care Committee (ACC). Committee functions are administrated through the Office of Animal Care and Institutional Biosafety (OACIB) within the Office of the Vice Chancellor for Research. All procedures are within guidelines established by the NIH for use of vertebrate animals and were approved by our institutional animal use committee prior to the execution of experiments. For all procedures with mice, they were anesthetized with halothane. All methods for euthanasia are consistent with recommendations of the NIH, the American Veterinary Medical Association and have been approved by our institutional animal use committee (ACC). For all experiments, animals were first anesthetized with halothane, and then sacrificed by decapitation on a guillotine without being allowed to regain consciousness. In all cases, tissues were removed for analysis after sacrifice.

### Antibodies and reagents

In this work we used the following antibodies: 63–90 [35] and H2 clones are monoclonal antibodies against (mAb) kinesin-1 light chains (KLCs) [27, 34] and kinesin-1 heavy chains (KHC) [36] respectively, TrkB, a rabbit polyclonal antibody from Santa Cruz Biotechnology Cat. # Sc-11. Mouse α-tubulin clone YL1/2 from Abcam. Mouse monoclonal antibody to tau protein clone PC1C6, MAB 3420 from Millipore. Protein kinase inhibitor DMAT was obtained from Calbiochem, diluted in DMSO or ethanol as appropriate and kept at -20°C until use. CK2 specific substrate was obtained from ANASPEC # 60537, active CK2 tetramer (α_2_β_2_) was from New England Biolabs.

### Atomic force microscopy

Peptide solutions were characterized using a Nano-Scope IIIa scanning probe work station equipped with a MultiMode head using a vertical engage E-series piezoceramic scanner (Veeco, Santa Barbara, CA). AFM probes were single-crystal silicon microcantilevers with 300-kHz resonant frequency and 42 Newton/meter spring constant model OMCL-AC160TS-W2 (Olympus). A 10μl of 0.1M NaOH was spotted onto mica, rinsed with 2 drops of deionized H_2_O, then a 10-μl sample solution of PrP_106-126_ or PrP-FL (From a 20μM stock solution) were spotted on freshly cleaved mica, incubated at room temperature for 3 minutes, rinsed with 20μl of filtered (Whatman Anotop 10) MilliQ water (Millipore), and blown dry with tetrafluoroethane (CleanTex MicroDuster III). Image data were acquired at scan rates between 1 and 2 Hz with drive amplitude and contact force kept to a minimum. Data were processed to remove vertical offset between scan lines by applying zero order flattening polynomials using Nanoscope software (Version 5.31r1,Veeco).

### Preparation of PrP solutions

Synthetic PrP peptides including PrP_**106-126**_ and control PrP_**106-126**_ scrambled (PrP-Scram, same amino acids as in PrP_**106-126**_ but in scrambled order) were synthesized at the University of Illinois at Chicago Research Resources Center. PrP_**106-126**_ and PrP-Scram lyophilized PrP peptides (0.5 mg) were reconstituted in nuclease free deionized water at 4°C at a 1mM final concentration (stock), aliquoted into several 0.5ml centrifuge tubes, and stored at -80°C until use. Recombinant PrP-FL was obtained from Dr. Jiyan Ma [5, 14, 37]. Before treating cells in culture or perfusing axoplasm at 2μM concentration with any PrP construct, 1 mM PrP stock aliquots were incubated at 37°C for 1 hour. Atomic force microscopy analysis of PrP-FL and PrP_106-126_ peptides in solution revealed an oligomeric tertiary conformation (see S4 Fig).

### Lysate preparation and immunoblot analysis

Cell cultures were homogenized in ROLB buffer (10mM HEPES buffer (pH 7.4), 0.5% Triton X-100, 80mM β-glycerophosphate, 50mM sodium fluoride, 2mM sodium orthovanadate, 100nM staurosporine, 100nM K252a, 50nM okadaic acid, 50nM microcystin, 100mM potassium phosphate and mammalian protease inhibitor cocktail [Sigma]), lysates were clarified by centrifugation and proteins were separated by sodium dodecyl sulfate polyacrylamide gel electrophoresis (SDS-PAGE) on 4–12% Bis-Tris gels (NuPage minigels, Invitrogen), using Mops Running Buffer (Invitrogen) and transferred to polyvinylidene fluoride (PVDF) membranes as previously described [38]. Immunoblots were blocked with 5% nonfat dried milk, in phosphate-buffered saline, pH 7.4, and probed with appropriate polyclonal or monoclonal antibodies. When phosphorylation sensitive antibodies were used, 50mM sodium fluoride was added to the blocking and primary antibody solutions to prevent dephosphorylation. Primary antibody binding was detected with horseradish peroxidase-conjugated anti-mouse and anti-rabbit secondary antibody (Jackson Immunoresearch) and visualized by chemiluminescence (ECL, Amersham). For relative quantification the level of immunoreactivity was determined by measuring the optical density (average pixel intensity) of the band that corresponds using ImageJ software (ImageJ 1.42q,NIH, http://rsbweb.nih.gov/ij). Isolation of membrane vesicle fractions from axoplasms was done as described before [39]. Two axoplasms from the same squid were prepared and incubated with appropriate effectors (PrP_**106-126**_ in perfusion buffer or perfusion buffer alone) and vesicle fractions evaluated by immunoblot using H2 and Trk antibodies. Trk served as protein loading control and vesicle fraction marker.

### Motility studies in isolated squid axoplasm

Axoplasms were extruded from giant axons of the squid, *Loligo pealeii*, at the Marine Biological Laboratory (MBL) as described previously [36, 40–42]. Squid axoplasms were extruded at the Rowe building of the MBL (Woods Hole, MA). Squid were handled in accordance with procedures dictated by the MBL Laboratory Animal Facility. Our laboratory located at the MBL has the proper authorization from the manager of the Marine Resources Department at MBL for the housing and euthanasia of squid. The MBL Laboratory Animal Facility is a USDA registered, and the MBL has an approved animal welfare assurance (A3070-01) from the Office for the Protection of Research Risks. The constitution of the Institutional Animal Care and Use Committee (IACUC) is in accordance with USPHS policy. In brief, a healthy translucent squid of approximately 30 cm in length is held by its mantle and the head severed above its eyes using a scissors followed immediately by destruction of the brain without sedation [43]. The mantle is cut open along the midline and the viscera and pen are removed carefully to avoid damaging the giant axons. The fins are removed with scissors and peel the skin off with tissue forceps. Identify the pair of axons lying parallel to the midline on each side of the open mantle. Dissect both axons very carefully to avoid touching the giant axons as it may damage the axolemma. Tie off the proximal end of the giant axon (near the stellate ganglion) and distal end with two different color cotton thread to help assure the orientation of the axons. Once both extremes are tied off tight, cut the giant axons 5 mm away from the knots to release the giant axons (pair of sister axoplasms). Gently tease away any connective tissue with extreme care not to damage the axonal membrane. Place the axon on a coverslip and cut the proximal end (white thread) hold the axon by the black thread and press the polyethylene tube near the distal end (black thread). Pull the axon steadily by the black thread to extrude the axoplasm. Then place spacers on both sides of the extruded axoplasm and place a coverslip on top without shearing the axoplasm to create a chamber where to perfuse the axoplasm with the effectors diluted in buffer X/2. Extruded isolated axoplasms were 400–600 mm in diameter and provided approximately 5μl of axoplasm. Synthetic PrP peptides and recombinant full length PrP (PrP) and inhibitors were diluted into X/2 buffer (175 mM potassium aspartate, 65 mM taurine, 35 mM betaine, 25 mM glycine, 10 mM HEPES, 6.5 mM MgCl2, 5 mM EGTA, 1.5 mM CaCl2, 0.5 mM glucose, pH 7.2) supplemented with 2–5 mM ATP; 20 ml of this mix was added to perfusion chambers. Preparations were analyzed on a Zeiss Axiomat with a 100, 1.3 NA objective, and DIC optics. Hamamatsu Argus 20 and Model 2400 CCD camera were used for image processing and analysis. Organelle velocities were measured with a Photonics Microscopy C2117 video manipulator (Hamamatsu) as described previously [44]. Approximately 20 squids were sacrificed.

### Live imaging analysis of mitochondria axonal transport

Hippocampal neurons from 3 DIV cultures were transfected (Lipofectamine 2000, Invitrogen) with a plasmid encoding a yellow fluorescent protein attached to a mitochondrial targeting sequence (mitoYFP, OriGene) to allow *in vivo* organelle visualization. 4 hours after transfection, cultures were treated as indicated in each case and placed on a recording chamber at 37°C and 5% CO2 with phenol red-free Neurobasal medium (Gibco). Time-lapse images of axonal mitochondria were acquired in an Olympus IX81 inverted microscope equipped with a Disk Spinning Unit (DSU), epifluorescence illumination (150W Xenon Lamp) and a microprocessor. Fast image acquisition was achieved with a 60X oil immersion objective and an ORCA AG (Hamamatsu) CCD camera. Time-lapse images were recorded over 10 min, at a rate of 1 frame every 3 sec. Mitochondrial movement was analyzed visually with the Multi Kymograph plugin of Fiji (http://fiji.sc/) and by counting the proportion and direction of fragments that move for more than 3 μm over an axonal segment of 30μm. We consider axons, the major processes, to be those processes that were at least 40–50 μm longer that any other process in a given hippocampal neuron. Typically the axons we measured were between 120–150 μm in length. To confirm the identity of axonal processes we stained 3 DIV hippocampal neurons with an antibody against the axonal resident protein Tau (tau-1) and alfa tubulin (S1 Fig). We consider the movement towards the tip of the axon the anterograde direction and the movement towards the cell body the retrograde direction. Instantaneous velocities of mobile mitochondria was calculated over 3 frames during 10 seconds in the anterograde and retrograde direction. Data correspond to three independent experiments per condition.

### Purification of membrane vesicle fractions from squid axoplasms by lodixanol vesicle flotation assay

After incubating axoplasms with appropriate effectors (10μM Prion _**106-126**_ or PrP scrambled) for motility assays in X/2 buffer plus 1mM ATP in 25μl final volume, after 50 minutes the axoplasms were moved to a low-protein binding 1.5ml centrifuge tube containing 200μl of homogenization buffer [10mM HEPES, pH7.4, 1mM EDTA, 0.25M sucrose, 1/100 protease inhibitor cocktail for mammalian tissue (Sigma; No. P8340), 1/100 phosphatase inhibitor cocktail set II (Calbiochem; No. 524627), 2sM K252a, and 1μ PKI], and homogenized by three passages through a 27G needle and two passages through a 30G needle attached to a 1ml syringe. Axoplasm homogenates were adjusted to 30% iodixanol by mixing 200μl of axoplasm homogenates with 300μl of solution D (50% (*w/v)* Iodixanol (Sigma), 10mM MgCl2, 0.25M sucrose). A 500μl layer of solution E (25% (*w/v)* Iodixanol, 10mM MgCl2, 0.25M sucrose) was gently loaded on top of the lysate adjusted to 30% Iodixanol, followed by a 100il layer of solution F (5% (*w/v)* Iodixanol, 10mM MgCl2, 0.25M sucrose. Samples were centrifuged at 250,000g for 30 minutes at 4°C in RP55S Sorval rotor. Following the centrifugation, 200μl was removed from top, which contained the vesicles/membranes and transferred to a new 1.5ml centrifuge tube. 1.2ml cold methanol was added and incubated on ice for 60 minutes, centrifuged at 14,000RPM in a tabletop centrifuge for 30 minutes. We resuspended the precipitated vesicles/membrane fraction pellets in 40μl of 1% SDS using orbital rotor for 1 hour at 300RPM. 10μl of 6x Laemmli buffer was added, and 15μl of each sample was analyzed by immunoblotting.

### CK2 *in vitro* kinase assay

The i*n vitro* kinase assay mixture contained in a 50μl final volume: 100μM CK2 synthetic R_3_A_2_D_2_SD_5_ peptide, 2U (1.05ng) CK2αβ from NEB Cat# P6010S, 1X reaction buffer (20mM Tris-HCl, 50mMKCl, 10mM MgCl_2_, pH 7.5 at 25°C) 100μM cold ATP containing 1.5mCi [γ^32^P] ATP; 1Ci = 37 GBq, and brought to a final 50μl with 20mM hopes, pH 7.4. We added the different PrP constructs (PrP-FL and PrP_**106-126**_) at 2μM final concentration. Incubation was carried out for 20 minutes at 30°C. Reactions were stopped by the transfer of 10μl of the reaction to P81 phosphocellulose circles and washed three times in 75mM phosphoric acid, dried, and analyzed by scintillation counting.

### Statistical analysis

Statistical comparisons were obtained by using GraphPad Prism 6 software. All experiments were repeated at least three times, using different brain specimens, extruded axoplasms or cell cultures derived from embryos from at least three different rat or mice and at least 3 different axoplasms. Data represents mean ± SEM. Mean differences were considered significant at the p ≤ 0.05. Multiple group comparisons were performed by one-way ANOVA with post-hoc Tukey. For pair comparisons, Student’s t-tests were used.

## Results

### PrP inhibits fast axonal transport

**Fig 1:**
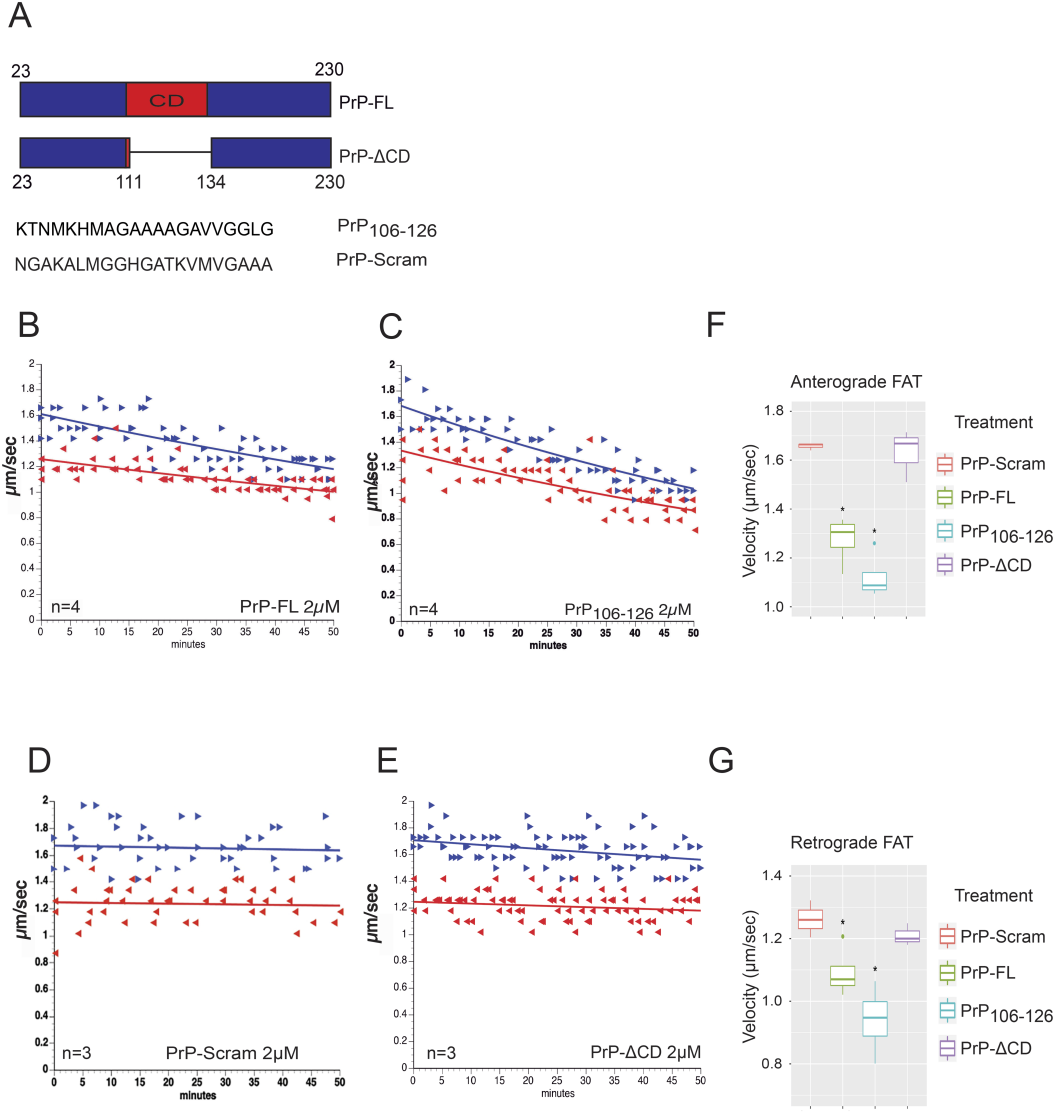
Full length PrP (PrP-FL) inhibits fast axonal transport of membrane-bounded organelles in isolated squid axoplasm. (**A**) Schematic representation of different PrP constructs and peptides used in this work. The PrP central domain (CD) is indicated in the top graph in red. Note that the truncated PrP (PrP-ΔCD) lacks most of the PrP CD. Two PrP peptides of 21 amino acids were used, one that corresponds to the amino acids 106 to 126 (PrP_**106-126)**_, and the other is the corresponding scrambled control peptide (PrP-Scram). Plots in **B, C, D** and **E** represent results from vesicle motility assays in isolated extruded squid axoplasms perfused with different PrP constructs. Blue arrowheads and blue line represent fast axonal transport (FAT) rates of kinesin-1 driven vesicles moving in the anterograde direction and the red arrows and red lines represent retrograde dynein-mediated FAT rates. Lines represent the best fit exponential of rates for vesicles moving in the anterograde blue arrows and retrograde red arrows directions over time in axoplasms. (**B**) Perfusion with 2μM of PrP-FL showed a marked reduction of anterograde and retrograde FAT soon after perfusion, compared to perfusing X/2 buffer alone [48] (data not shown in this manuscript). (**C**) Perfusion of a PrP full length construct lacking amino acids 111 to 134 (PrP-ΔCD) showed not effect on FAT **(D)** Perfusion with PrP_**106-126**_, a 21 amino acid peptide corresponding to the PrP CD inhibited bidirectional FAT with a profiles of inhibition almost identical to the one induced by PrP-FL. (**E**) Perfusion of the PrP_**106-126**_-Scram control peptide encompassing the same amino acids but arranged in a scrambled order did not alter FAT. Graphs showing quantitation of average rates of anterograde (**F**) and retrograde (**G**) FAT obtained 30–50 minutes after PrP perfusion indicating that when PrP-FL and its 21 amino acid peptide corresponding to the central domain of PrP-FL are perfused they induce bidirectional FAT inhibition. Letter “n” represents the number of independent axoplasms perfused per construct. Light blue and green dots in graphs F and G represent outlier values.

Several reports document FAT deficits in animal models of prion diseases, consistent with the dying back pattern of degeneration observed in these diseases [11, 24, 25, 45, 46]. Various neurotoxic effects were associated with intracellular accumulation of wild type, non-infectious PrP-FL [5], but whether PrP-FL could directly affect FAT was not previously tested. Towards this end, we performed vesicle motility assays in isolated squid axoplasms. By using video-enhanced contrast DIC microscopy, the isolated axoplasm preparation allows for accurate quantitation of anterograde (conventional kinesin-dependent) and retrograde (cytoplasmic dynein-dependent) FAT rates [40, 47]. Because the plasma membrane is removed from the axon, both recombinant forms of PrP and PrP-derived synthetic peptides (Fig 1A) can be perfused into the axoplasm and their effect on FAT directly evaluated [40].

Perfusion of PrP-FL protein in axoplasm (2μM) triggered a significant reduction in both anterograde and retrograde FAT (Fig 1B), and a similar inhibitory effect was also observed when PrP-FL was perfused at much lower concentration (PrP-FL 100nM, S2 Fig). This finding prompted us to map specific PrP-FL domains mediating the toxic effect. The positively charged central domain (CD, amino acids 94–134) has been shown to play a role in the neurotoxic effects elicited by pathogenic forms of PrP [49–51]. Li and coworkers showed that the PrP residues 105–125 may constitute a neurotoxic functional domain [49]. Furthermore, Simoneau and coworkers determined that the 106–126 hydrophobic domain at the surface of oligomeric full length PrP was essential for toxicity [12]. Extending these findings, experimental data documented toxic effects of a PrP peptide encompassing residues 106–126 on primary hippocampal, cortical and cerebellar cultured neurons [52–57]. Together, these findings prompted us to evaluate whether the CD domain may mediate the toxic effect of PrP-FL on FAT. Consistent with this possibility, recombinant PrP-ΔCD did not affect FAT when perfused in axoplasm (Fig 1C). Further, a synthetic peptide comprising amino acids 106–126 of PrP (PrP_**106-126**_) triggered a dramatic inhibition of FAT (Fig 1D), whereas a scrambled version of this peptide (PrP-Scram) did not (Fig 1E). Quantitative analysis of FAT average rates obtained from 30–50 minutes after perfusion demonstrated a significant reduction in both anterograde and retrograde FAT rates induced by PrP-FL and PrP_**106-126**_, but not by control PrP-Scram or PrP-ΔCD (Fig 1F and 1G, and S1 Table). Collectively, these experiments indicate that PrP-FL inhibits FAT, and that the CD of PrP^c^ is both necessary and sufficient to trigger this toxic effect.

### PrP induces alterations in mitochondrial axonal transport

**Fig 2:**
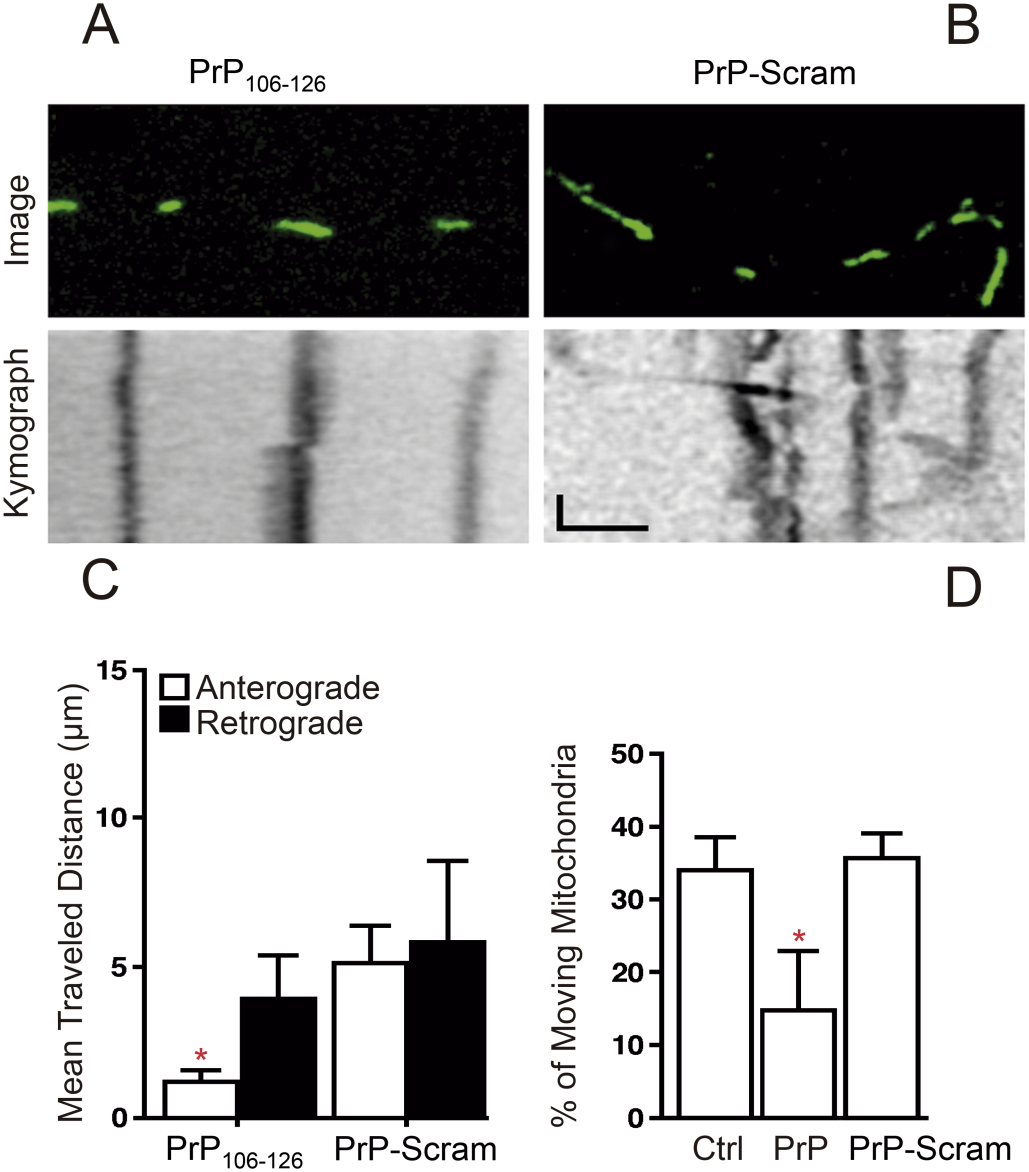
Prion inhibits fast axonal transport of mitochondria in mammalian cultured neurons. The effects of Prion on mitochondria mobility was analyzed in 3 days *in vitro* rat embryonic primary hippocampal neurons by time-lapse microscopy. (**A**) Upper panel shows fluorescently labeled mitochondria from axons of neurons treated with PrP_**106-126**_ or control PrP_**106-126**_-Scram. In the lower panel, kymographs reveal the trajectory of mitochondria motility from neurons incubated for 1 hour with 3μm PrP_**106-126**_ versus PrP_106-126_-Scram control peptide (**B**). Kymographs were obtained from images in the upper panel. Scale bar in the X-axis equals 30μm and in the Y-axis equals 60 seconds. (**C**) Quantification of the distance traveled by mitochondria analyzed in (**B)** in the anterograde (white) and retrograde (black) direction. (**D**) Quantification of the percentage of moving mitochondria in neurons treated with 3μm PrP_**106-126**_ compared to control PrP_**106-126**_-Scram, or non-treated control neurons (Ctrl). (**C-D**) Mean ±SEM, * p<0.05, total of 26 neurons were analyzed, 7 (PrP-Scram treated), 8 (Control un-treated), 11 (PrP_**106-126**_ treated). A total of 143 mitochondria were analyzed in figures C and D; 57 mitochondria were analyzed in scramble treated neurons 26 (Not mobile), 12 (anterograde direction), 19 (retrograde direction); 86 mitochondria were analyzed in PrP_106-126_ treated neurons, 58 (not mobile), 7 (anterograde direction), 21 (retrograde direction). Results were obtained from 3 independent experiments. One-way ANOVA with post-hoc Tukey.

Based on results from experiments in Fig 1, we next evaluated whether PrP alters FAT of mitochondria in mammalian cultured neurons. Because labeling of mitochondria with Mito Tracker Red or tetramethylrhodamine ethyl ester dyes can interfere with mitochondrial mobility [58, 59], we transfected primary mouse embryonic hippocampal neurons in culture with a plasmid encoding a mitochondrial resident protein fused with yellow fluorescent protein (mito-YFP). At day 3 *in vitro* (3 DIV), we incubated transfected neurons for one hour with 3μM PrP_**106-126**_ (Fig 2A) or with control PrP-Scram (Fig 2B), and analyzed mitochondrial motility for 10 minutes using time-lapse microscopy.

Consistent with the marked reduction of FAT observed in axoplasms treated with PrP_**106-126**_ peptide, kymograph analysis revealed a marked reduction of mitochondria mobility in neurons treated with PrP_**106-126**_ (Fig 2A), compared to neurons treated with PrP-Scram (Fig 2B). Specifically, the average distance traveled in the anterograde direction was significantly reduced in PrP_**106-126**_ (1.22± 0.33μm) compared to PrP-Scram treated cell (5.17± 1.23μm) (Fig 2C). Similarly, the percentage of motile mitochondria in either direction was significantly reduced in PrP_**106-126**_ (14.79±8.05) versus PrP-Scram treated cell (35.56± 3.32) or untreated control cells (35.88± 4.47) (Fig 2D). Similarly, instantaneous velocities in the anterograde and retrograde directions were also evaluated. Time-lapse microscopy revealed that PrP_106-126_ decreces mitochondria instantaneous velocity in the anterograde direction (S5 Fig). These results extended findings of PrP_**106-126**_ toxicity in isolated squid axoplasm to mammalian cultured neurons, further revealing alterations in FAT of mitochondria.

### The protein kinase CK2 mediates PrP-induced FAT inhibition

**Fig 3:**
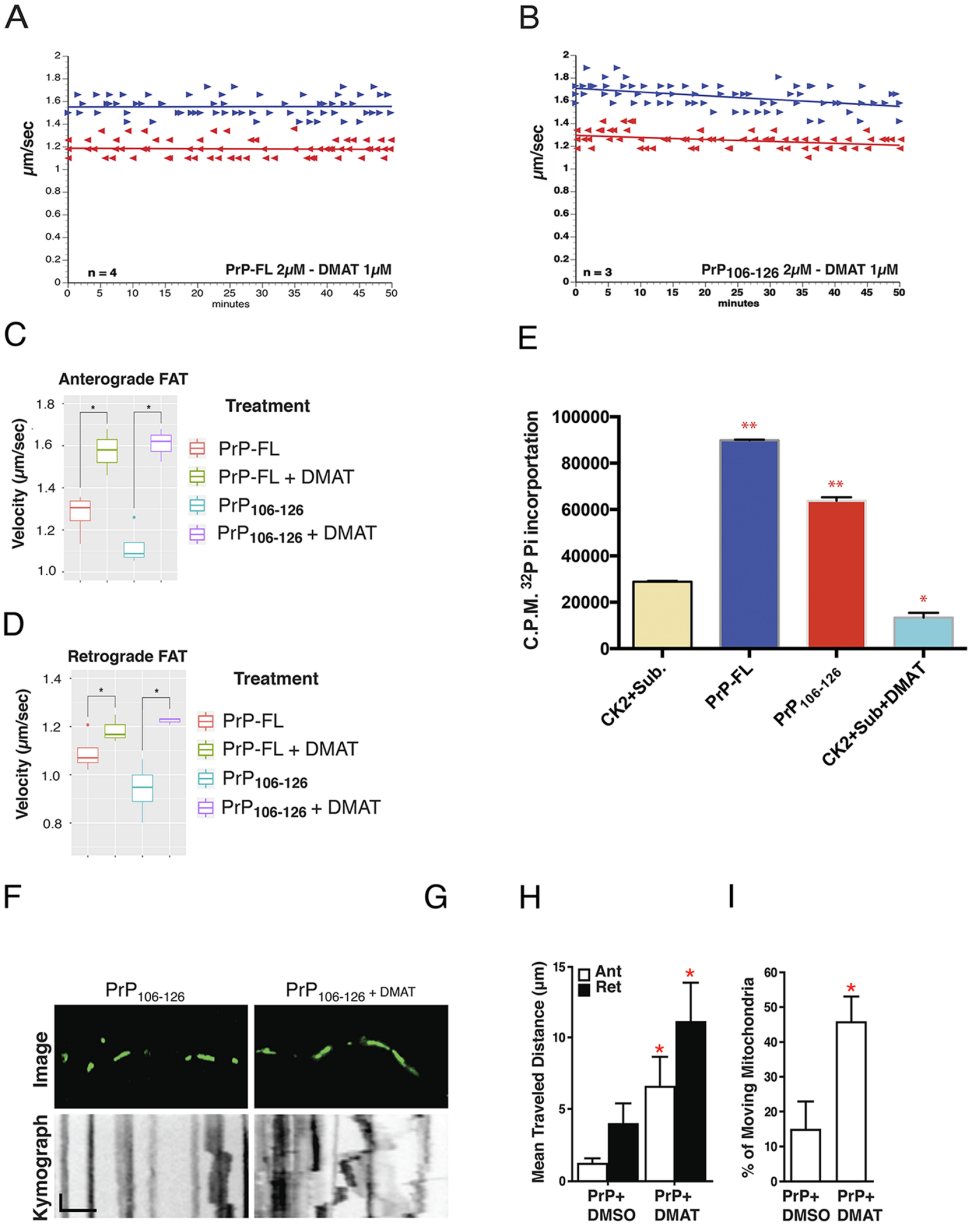
Casein kinase 2 mediates PrP-induced fast axonal transport inhibition. Plots in A-B depict results from vesicle motility assays as in Fig 1. Co-perfusion experiments of PrP-FL with DMAT, a highly specific CK2 inhibitor, prevent bidirectional FAT inhibition (Compare with Fig 1A). (**B**) Similarly, co-perfusion of PrP_**106-126**_ with DMAT prevents the inhibitory effect of PrP_**106-126**_ on FAT (Compare with Fig 1D). Graphs showing quantification of average rates of anterograde (**C**) and retrograde (**D**) FAT obtained 30–50 minutes after co-perfusion of PrP-FL and PrP_**106-126**_ with DMAT indicated that CK2 plays a key role in PrP-induced FAT inhibition. (**E**) The stimulatory effect of PrP-FL and PrP_**106-126**_ peptide on CK2 activity was evaluated *in vitro* by CK2 kinase assay as described in the experimental section. Bar chart of the phosphorylation kinase activity of CK2 expressed as the incorporation of radioactive inorganic phosphate (P_i)_ into the synthetic R_3_A_2_DSD_5_ peptide by the recombinant CK2ed in the experimental section. Bar chart of the phosphorylation kinase activity of CK2 expressed as the incorp_**106-126**_ (Red bar). These results suggest that PrP can activate CK2 directly. C.P.M. stands for counts per minute in arbitrary units. Scintillation counting-based quantitation from 3 independent experiments. *: p<0.0002; **: p<0.0001. Two-tailed P values. (**F**) Upper panel shows fluorescently labeled mitochondria from axons of 3 DIV neurons treated with either PrP_**106-126**_ or control PrP_**106-126**_-Scram peptides. In the lower panel, kymographs reveal the trajectory of mitochondria motility from neurons incubated with 3μm of either PrP_**106-126**_ or (**G**) PrP_**106-126**_ plus 5μM DMAT for 1 hour. (**H**) Quantification of average distance traveled by mitochondria as analyzed in (F) and (G) in the anterograde (white) and retrograde (black) direction. Note the lack of inhibitory effect when neurons are co-incubated with 5μM DMAT. (**I**) Quantification of the percentage of mobile mitochondria analyzed in (F) or (G). Note a reduction of mobile mitochondria in PrP_**106-126**_ and the dramatic recovery of mitochondria mobility in PrP_**106-126**_ plus 5μM DMAT co-treated neurons. These pharmacological results suggest that the activation of endogenous axonal CK2 may be responsible for the inhibition of both directions of FAT induced by cellular PrP. (**H-I**) Mean ±SEM, * p<0.05, total of 22 neurons were analyzed, 11 (PrP_**106-126**_ treated) and 11 (PrP_**106-126**_ + DMAT treated). Results were obtained from 3 independent experiments. One-way ANOVA with post-hoc Tukey.

Several phosphotransferases have been identified that regulate FAT by modifying functional specific motor protein subunits [60–63]. Among protein kinases tested in the isolated axoplasm preparation, casein kinase 2 (CK2) inhibited FAT with an inhibitory profile similar to that induced by PrP-FL and PrP_**106-126**_ (Fig 1B and 1C) [27], prompting us to evaluate whether the inhibition of FAT induced by PrP-FL or PrP_**106-126**_ was mediated by CK2. To this end, we co-perfused PrP-FL and PrP_**106-126**_ with Dimethylamino- 4,5,6,7-tetrabromo-1H-benzimidazole (DMAT), a highly specific and powerful ATP-competitive CK2 inhibitor [64] that effectively inhibits CK2 activity in the axoplasm preparation [27]. Remarkably, co-perfusion of either PrP-FL or PrP_**106-126**_ with DMAT completely prevented the inhibitory effect on FAT (Fig 3A and 3B). Quantitation of average FAT rates 30 to 50 minutes after perfusion confirmed that activation of endogenous CK2 mediates the inhibitory effects of both PrP-FL and PrP_**106-126**_ on FAT (Fig 3C and 3D and S1 Table).

Interestingly, these results were consistent with prior reports showing that PrP interact with CK2 and modulate its activity [32, 33]. To evaluate whether PrP-FL could directly activate CK2, we conducted *in vitro* kinases assays using recombinant CK2 and a highly specific CK2 peptide substrate R_3_A_2_DSD_5_ as radioactive phosphate acceptor [65]. Remarkably, PrP-FL induced significant activation of CK2 tetramer (Fig 3E), and similar activation was triggered by PrP_**106-126**_ (Fig 3E), suggesting that the inhibitory effect of PrP-FL and PrP_**106-126**_ on FAT may result from directly activating CK2.

Next, we evaluated whether CK2 mediated the inhibition of mitochondria mobility induced by PrP_**106-126**_ in mammalian neurons. To this end, we simultaneously treated primary neurons in culture with both PrP_**106-126**_ (Fig 3F) and the CK2 inhibitor DMAT (2μM; Fig 3G) and measured mitochondrial mobility as done in Fig 2. Kymograph analysis showed a consistent increase of mitochondria mobility for neurons co-incubated with PrP106_**-126**_ plus DMAT (Fig 3G **lower panel**) compared to neurons treated with PrP_**106-126**_ and DMSO vehicle (Fig 3F **lower panel**). As expected, treatment of neurons with DMAT alone did not alter mitochondria FAT (S3 Fig). Quantitative analysis confirmed that average distances traveled by individual mitochondria in either anterograde (6.56±2.09μm) or retrograde (11.05±2.74μm) direction were significantly higher in neurons co-treated with DMAT compared to average distances of individual mitochondria from PrP_**106-126**_ treated neurons (anterograde: 1.21±0.38μm; retrograde 3.97±1.44μm) (Fig 3H). Additionally, the percentage of moving mitochondria (Fig 3I) was significantly higher in DMAT-treated neurons (45.51±7.29μm) than in PrP_**106-126**_ treated neurons (14.72±8.17μm). Collectively, results from these experiments indicated that the inhibitory effects of PrP-FL and PrP_**106-126**_ on FAT are mediated by CK2.

### PrP-induced CK2-activation promotes conventional kinesin phosphorylation and release from vesicular cargoes

**Fig 4:**
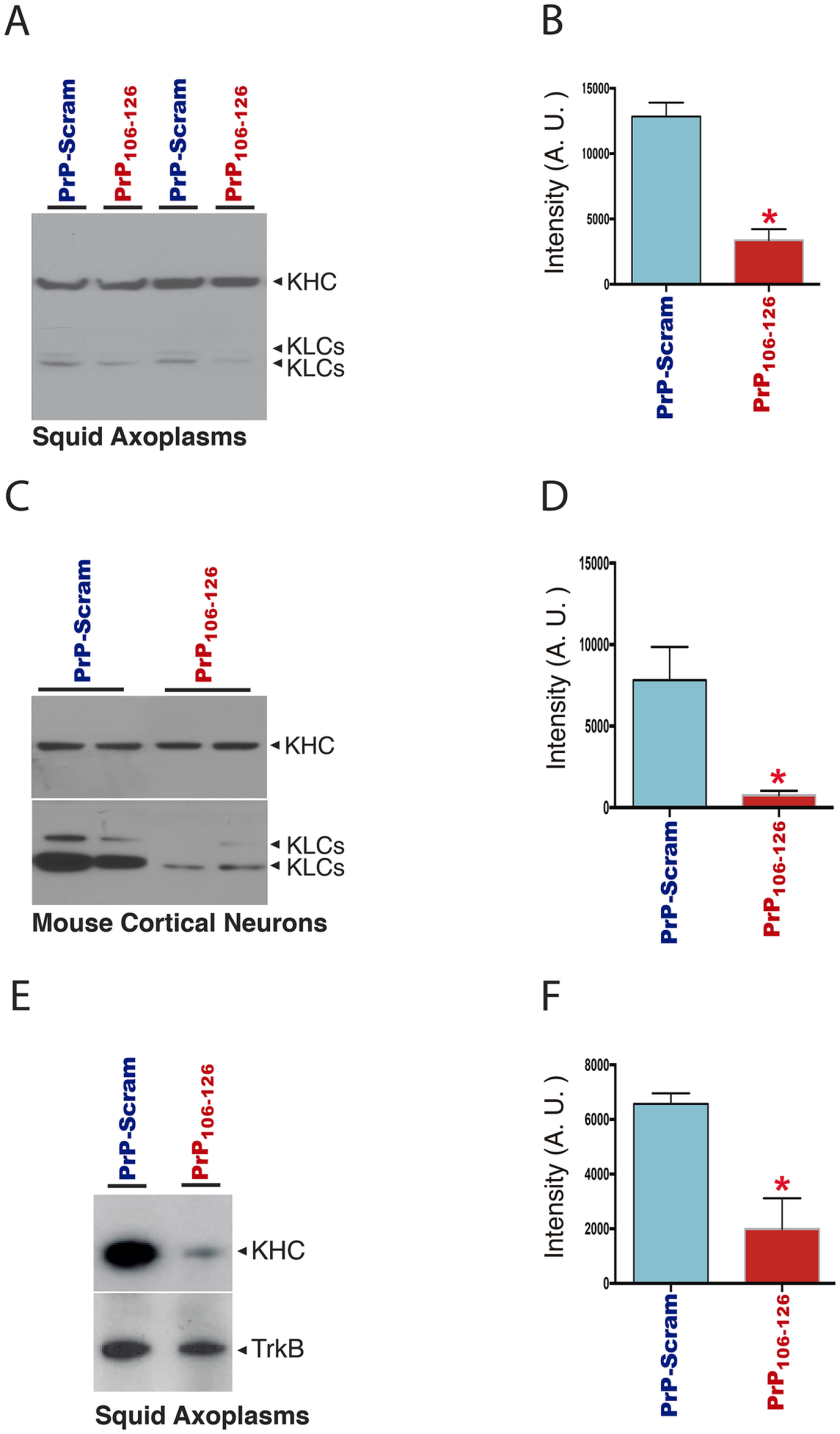
PrP induces CK2-mediated phosphorylation of kinesin light chain subunits and detachment of conventional kinesin from membrane cargoes. (**A-B**) Quantitative immunoblot analysis of kinesin-1 from two pair of sister axoplasms incubated either with control PrP-Scram or PrP_**106-126**_ peptides and (**C-D**) primary embryonic mouse cortical neurons cultured for 3 days in vitro treated for one hour. Antibody 63–90 preferentially recognizes kinesin-1 light chains (KLCs) when they are not phosphorylated by CK2 [27, 35]. (**B and D**) Quantitation graph bars show that PrP_**106-126**_ decreased the immunoreactivity for 63–90 versus control PrP-Scram treated axoplasms and neurons respectively. Note a significant reduction (**B**) of immunoreactivity when neurons were treated with PrP_**106-126**_ compared to PrP-Scram; (n = 5, number of independent experiments. p = 0.0313, significance was assessed at P < 0.05). (**D**) Significant reduction of 63–90 immunoreactivity in axoplasms incubated with PrP_**106-126**_ compared to control PrP-Scram, (n = 3; number of independent experiments. p = 0.0355, significance was assessed at P < 0.05). (**E**) Vesicles purified from sister axoplasms by vesicle flotation assays perfused with control PrP-Scram and PrP_**106-126**_ synthetic peptide were assayed by Western blot for KHC and TrkB. TrkB was used as membrane protein marker and for loading control. (**F**) Quantitation graph bars shows a significant reduction of kinesin-1 association to purified vesicles in PrP_**106-126**_ incubated extruded axoplasms compared to control PrP-Scram treated axoplasms, (n = 3, number of independent experiments; significance was assessed at P < 0.05). Taken together, these experiments suggest that PrP_**106-126**_ increases the intracellular activity of CK2, which in turn results in KLCs phosphorylation and kinesin-1 release from its cargo vesicles.

CK2 has been shown to directly phosphorylate kinesin light chain (KLCs) subunits of the major motor protein conventional kinesin [27, 66–69]. Based on that precedent and on results from experiments in Fig 3, we evaluated whether PrP toxicity involves alterations in KLC phosphorylation. To this end, we perfused axoplasms with PrP_**106-126**_ (Fig 4A and 4B) and also incubated primary hippocampal neurons with PrP_**106-126**_ (Fig 4C and 4D).

KLC subunit phosphorylation was evaluated by semi-quantitative Western blotting using 63–90, an antibody that preferentially recognizes a dephosphorylated CK2 epitope on KLCs [27, 70]. A significant reduction in 63–90 immunoreactivity was observed in both perfused squid axoplasms (Fig 4A) (approx. 74% reduction, Fig 4B) and cultured neurons (Fig 4C) (approx. 90% reduction, Fig 4D) incubated with PrP_**106-126**_. Since 63–90 immunoreactivity is reduced by KLC phosphorylation [27, 34, 60], immunoblotting results suggest that PrP_**106-126**_ promotes increased KLC phosphorylation (Fig 4A–4D).

**Fig 5:**
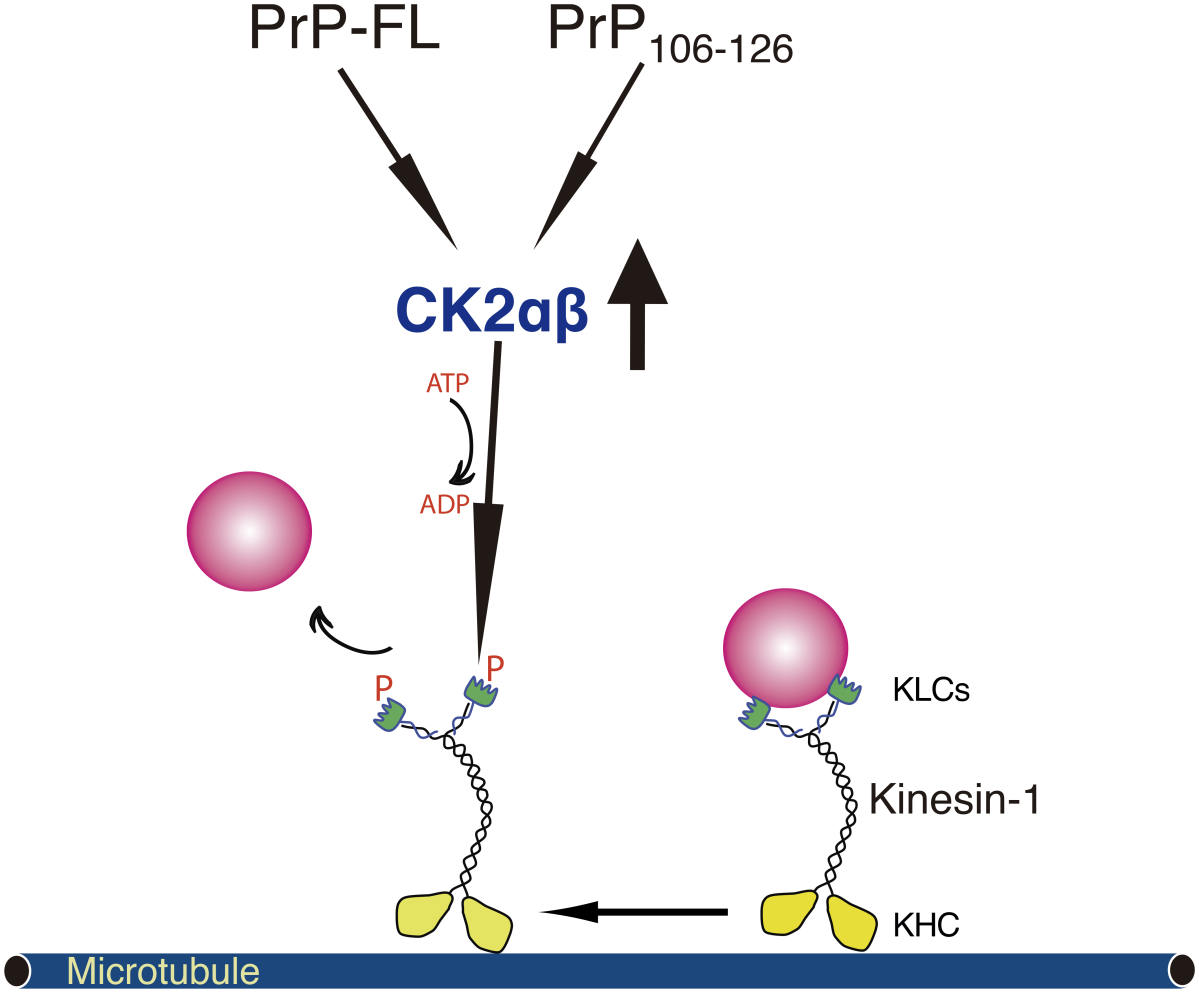
Proposed molecular mechanism for PrP-induced FAT inhibition. Pharmacological data showed here indicates that PrP-induced FAT inhibition is mediated by activation of endogenous tetrameric CK2αβ and subsequent phosphorylation of KLCs. Phosphorylation of KLCs (red letter P) promotes the detachment of conventional kinesin from its transported vesicular cargoes. Our experimental data suggests that both PrP-FL and its central domain (CD) peptide of 21 amino acids PrP_**106-126**_ inhibit FAT with an identical profile of inhibition.

CK2-mediated phosphorylation of KLCs reportedly promotes detachment of transported MBO cargoes [27, 34, 70–72]. Based on these precedents, we isolated vesicle fractions from axoplasms perfused with PrP_**106-126**_ or with PrP-Scram peptides (Fig 4E). Levels of conventional kinesin subunits associated with vesicles was evaluated by immunoblotting using H2 monoclonal antibody against kinesin heavy chain (KHC) subunits and normalized to anti-trkB-immunoreactive bands, as before [27, 39]. Consistent with our previous report showing CK2-dependent release of conventional kinesin from transported MBOs [27, 35, 71, 73, 74] KHC levels were reduced approximately 70% in membrane fractions prepared from squid axoplasms perfused with PrP_**106-126**_, compared to membranes prepared from PrP-Scram-perfused ones (Fig 4F). Together, these experiments indicated that PrP-induced FAT inhibition involves abnormal activation of endogenous CK2, phosphorylation of KLCs and a concomitant release of conventional kinesin from transported MBOs (Fig 5).

## Discussion

The molecular basis for prion disease (PrD) pathology remains unclear. Although the infectious form of PrP has received the most attention [75], prion infection is quite infrequent in humans. The majority of human cases (99%) are associated with mutations in the gene encoding PrP or occur sporadically [4]. In cases where PrP^Sc^ conformation is not required to induce pathogenesis, genetic and experimental studies suggest that the spontaneous accumulation of either mutant or wild type PrP can induce neuronal dysfunction and toxicity [4, 10, 76–78]. In addition, aggregated, non-infectious oligomeric PrP has also been shown to induce neurotoxicity without involving PrP^Sc^ [4, 9, 11, 12]. Atomic force microscopy-based structural analysis of PrP-FL and PrP_**106-126**_ indicate that both PrP constructs present a globular or oligomeric conformation (S4 Fig). Furthermore, Chiesa and collaborators demonstrated that 5 to 10-fold overexpression of wild type PrP can cause neuronal dysfunction and synaptic abnormalities through an aggregated, non-infectious PrP species [11]. Results from multiple laboratories indicate that cytosolic accumulation of full-length cellular PrP (PrP-FL) suffices to induce progressive neuronal toxicity and severe ataxia in cultured neurons and in living mice [5, 13–16]. This observed toxic phenomenon associated with increased dosage of PrP is not restricted to prion diseases, as other human progressive neuropathies including Alzheimer´s and Parkinson´s diseases are associated with aggregation of proteins induced by overexpression of wild type polypeptides [79–81]. Collectively, these observations suggest that a variety of molecular factors, including increased PrP dosage and conformation-dependent conversion of PrP to a neurotoxic species may underlie prion disease pathology, thus providing a common pathological framework for seemingly diverse prion disease variants.

There is no consensus on the specific cause of neuronal degeneration in PrD, but various pathological mechanisms have been postulated, including mitochondrial dysfunction and activation of neuronal apoptosis [82]. Although activation of apoptotic pathways will damage neurons in prion diseases [55, 83, 84], this is a generic explanation that does not provide insight into pathogenic mechanisms. Moreover, Chiesa and collaborators demonstrated that abolishing neuronal apoptosis in a transgenic model of familial prion disease effectively prevents neuronal loss, but does not prevent dying-back axonopathy and synaptic loss or delay the clinical symptoms [85]. These evidence suggests that, while apoptosis may be a component of prion diseases, changes in other vital neuronal processes may trigger the loss of synapses and clinical symptoms characteristic of these diseases.

Experimental evidence suggests that alterations in FAT might represent an early pathogenic event in prion diseases [45, 46, 85, 86] as well as other disorders linked to misfolded proteins [26, 73, 74, 87–91]. Sanchez-Garcia and co-workers showed that neurons expressing the PrP-M205,212S mutant form exhibit disrupted FAT and reduced synaptic accumulation of specific synaptic proteins important for axonal growth, vesicular fusion, secretion and neurotransmission [92, 93]. Similarly, Senatore and coworkers showed that mutant PrP suppressed neurotransmission in cerebellar granule neurons by altering the delivery of voltage-gated calcium channels [94]. Finally, Ermolayev and coworkers recently showed a direct and early link between prion clinical symptoms and FAT inhibition induced by different prion strains [24]. However, these studies did not provide mechanisms by which PrP affects FAT.

Data from Ma and collaborators showed that accumulation of full length PrP (PrP-FL) within the cytosol is neurotoxic *in vitro* and *in vivo* [5, 7]. Evidence from other groups showed that intracellular accumulation of wild type PrP leads to neuronal dysfunction and synaptic abnormalities [11, 56]. To test whether the neurotoxicity of intracellular PrP-FL was directly associated with FAT inhibition, we took advantage of the isolated squid axoplasm preparation. This unique *ex vivo* model facilitated the initial discovery of kinesin-1 [95], and kinase-based regulatory mechanisms for FAT [96, 97].

Here we present direct experimental evidence that PrP-FL, at physiologically plausible concentrations (100nM to 2μM), is a strong inhibitor of FAT. Previous work had mapped the toxicity of PrP primarily to the central domain (CD) [75, 98–100]. Consistent with these studies, the observed effects of PrP on FAT required the central domain (CD), as perfusion of a PrP construct lacking most of the central domain (PrP-ΔCD) does not affect FAT. Furthermore, perfusion of a cell permeable 20mer synthetic peptide corresponding to the positively charged CD (PrP_**106-126**_**)** [101] showed a toxic inhibitory effect comparable to that elicited by PrP-FL. Together these *ex vivo* experiments suggest that the PrP CD is required and sufficient to inhibit FAT.

Under normal physiological conditions, the concerted activity of kinases and phosphatases regulate FAT by controlling the functional activities of molecular motors [60–63]. Under pathological circumstances, misregulated signaling pathways can alter motor functions, leading consequently to altered FAT and dysfunctional synaptic transmission [26–28, 36, 39, 48, 67, 71, 73, 74, 87, 89, 102–108], harmful events that result in progressive synaptic dysfunction and dying back neuropathy. The ability of PrP^c^ to inhibit anterograde FAT at concentrations lower than of conventional kinesin [109] abrogates the possibility that PrP effects resulted from steric interference. Instead, alterations in regulatory signaling pathways for FAT appeared a more plausible mechanism.

Cell biological and pharmacological data shows that toxic PrP can affect the activity of various phosphotransferases capable of regulating FAT, including GSK3β [29], PI3K [30], and JNK [31], but do not indicate whether these changes are direct or indirect consequence of pathogenesis. Recent studies indicate that PrP can associate with and modulate CK2 activity [32, 33]. Here we showed that co-perfusion of inhibitors of CK2 with either PrP-FL or PrP_**106-126**_ completely abolishes the inhibitory effects of PrP on FAT. Although the CK2 inhibitor DMAT abolished the inhibition of mitochondria anterograde FAT induced by PrP, it also showed activation of retrograde FAT in combination with PrP_**106-126**_, an unexpected result that deserves future analysis. Although the precise mechanism by which PrP activates CK2 remains to be determined, *in vitro* CK2 kinase activity showed that recombinant PrP-FL and the synthetic peptide PrP_**106-126**_ are potent CK2 activators both *in vivo*, and *in vitro*, suggesting that the activation of CK2 may result from a direct interaction between PrP and CK2 *in vivo*.

Deficits in FAT of membrane-bounded organelles (MBOs) are responsible or at least significant contributors to multiple human neuropathies displaying dying back degeneration of neurons including hereditary spastic paraplegia, Alzheimer´s, Parkinson´s, Huntington´s, amyotrophic lateral sclerosis, and prion diseases [23, 24, 26, 27, 36, 73, 74, 87, 106, 107, 110–112]. Mitochondria are critical MBOs transported in neuronal cells, as these generate ATP needed for a wide variety of vital metabolic processes including FAT, neuronal growth, regeneration, and survival [113, 114]. A large body of experimental data documented deficits in FAT of mitochondria in various human neuropathies associated with altered kinase activities [73, 115–117]. Consistent with these reports, we showed a consistent reduction of mitochondria motility in PrP_**106-126**_ treated neurons versus PrP-Scram treated ones. Both the percentage of moving mitochondria and the average distance traveled were reduced. Although the regulatory mechanisms that govern the transport of membranous vesicles and mitochondria might not be the same [117] the effects were not restricted to mitochondria alone, as there was marked reduction in the bulk of vesicles moving in PrP-treated squid axoplasms. Interestingly, we observed a different profile of inhibition between mitochondria and squid axoplam vesicles. While PrP affected both directions of transport for these vesicles, it inhibited anterograde FAT to a greater extent than retrograde FAT for mitochondria, likely due to differences in the regulatory mechanisms between these MBOs. Consistent with evidence suggesting that PrP can activate CK2 both *in vitro* and *in vivo* and that activation of CK2 results in FAT inhibition, the potent and specific pharmacological CK2 inhibitor DMAT prevented PrP-induced FAT inhibition in both isolated axoplasm and in cultured neurons.

The remaining challenge was to determine how PrP and CK2 can compromise FAT. Previous studies had indicated that phosphorylation of KLCs by GSK3β and CK2 promotes release of conventional kinesin from MBOs and FAT inhibition [27, 34, 60]. Accordingly, biochemical experiments in this work revealed increased KLC phosphorylation in squid axoplasms and cultured mammalian neurons treated with PrP, as revealed by reduced immunoreactivity for 63–90, a monoclonal antibody that recognizes a CK2 dephosphoepitope within KLCs.

GSK3β and CK2-mediated phosphorylation of KLCs was shown to promote detachment of conventional kinesin from MBOs [27, 39, 60]. Consistent with these precedents, levels of kinesin-1 associated with axonal MBOs was significantly reduced in axoplasms perfused with PrP_**106-126**_, relative to PrP-Scram-perfused ones. Similar results were observed upon perfusion of recombinant CK2 and oligomeric amyloid beta (0Aβ), which induces endogenous CK2 activation [27]. That PrP-FL inhibited both anterograde and retrograde FAT suggests that CK2 may also affects cytoplasmic dynein [66] but further studies are needed to address this possibility.

Experiments in this work are in agreement with the idea that PrP-FL and its central peptide domain PrP_**106-126**_ inhibit FAT through a molecular mechanism involving abnormal activation of endogenous CK2, phosphorylation of KLCs, and release of conventional kinesin from transported MBO cargoes (Fig 5). In all likelihood, CK2 substrates other than molecular motors may contribute to prion pathology. CK2 has hundreds of reported substrates [118] many of which may contribute to axonal degeneration and synaptic loss in the context of prion disease [119]. Accordingly, oAβ-mediated increases in CK2 activity disrupt synaptic transmission and CK2 inhibitors restore it [105].

Results from this work demonstrate that pharmacological inhibition of CK2 prevents FAT inhibition induced by PrP. Thus, CK2 inhibition may represent a novel therapeutic intervention for prionopathies and other progressive neuropathies associated with abnormal CK2 activity and defects in FAT [27], [105], [120]. The recent development of blood-brain barrier permeable and highly selective CK2 inhibitors capable of accessing the brain makes this notion particularly compelling [121, 122]. Finally, Our results provide a basis for exploring the more complex pathology of a PrP transgenic mouse model in the near future.

## Supporting information

S1 Fig Mouse Hippocampal neuron cultured for 3 days showing the identity of minor processes and the axon. Confocal image showing a 3 days in culture hippocampal neuron immunostained with a monoclonal antibodies against (**A**) tyrosinated tubulin (tyr-Tub; green and green arrows) and (**B**) dephosphorylated Tau (Tau-1; red and red arrowhead). (C) superimposition of A and B. Note the axonal distribution of tau along the major process versus the widespread localization of tubular within the minor processes and cell body. Scale bar 20μm.
(TIF)

S2 Fig Full length PrP (PrP-FL) inhibits fast axonal transport. Plot represents results from vesicle motility assays in isolated extruded squid axoplasms perfused with PrP-FL at 100nM concentration. Blue arrowheads and blue line represent fast axonal transport (FAT) rates of kinesin-1 driven vesicles moving in the anterograde direction and the red arrows and red lines represent retrograde dynein-mediated FAT rates. Lines represent the best fit exponential of rates for vesicles moving in the anterograde blue arrows and retrograde red arrows directions over time in axoplasms. Perfusion with 100nM of PrP-FL showed a marked reduction of anterograde and a modest retrograde FAT soon after perfusion, compared to perfusing X/2 buffer alone [48] (data not shown in this manuscript) or PrP-Scram (Fig 1D).
(TIF)

S3 Fig The CK2 inhibitor DMAT does not alter FAT. Upper panel shows fluorescently labeled mitochondria from axons of 3 DIV neurons treated with either vehicle (DMSO) or the CK2 inhibitor DMAT 5μM for 60 min. In the lower panel, kymographs reveal the trajectory of mitochondria motility from neurons incubated with vehicle **(A)** or (**B**) 5μM DMAT for 1 hour. (**C**) Quantification of average distance traveled by mitochondria as analyzed in (**A**) 24.88±13.47 μm and (**B**) 20.76±5.16 μm in the retrograde direction. Note the lack of effect when neurons are incubated with 5μM DMAT alone compared to DMSO treated neurons. Scale bar in the X-axis equals 30μm and in the Y-axis equals 60 seconds. Mean ±SEM, total of 19 neurons were analyzed, 8 (Control DMSO treated) and 11 (DMSO+DMAT treated). Results were obtained from 3 independent experiments. One-way ANOVA with post-hoc Tukey.
(TIF)

S4 Fig Characterization of PrP-FL and PrP_106-126_ by atomic force microscopy analysis. AFM analysis showed a common oligomeric structure present in PrP-FL and PrP_106-126_. (**A**) Recombinant PrP-FL (1mM) and (**B**) synthetic PrP_106-126_ peptide resuspended in H_2_O at 1mM concentration were incubated at 37°C for 60 minutes. (**C**) Buffer x/2 alone. Solutions were diluted to 20μM with the same buffer used for perfusing squid axoplasms (Buffer X/2) and then analyzed on mica under ambient conditions using tapping mode AFM. White round dots in A-C seem to be attributed to salt in the X/2 buffer. All AFM images shown are 2_2-mm x–y, 10nm total z-range.
(TIF)

S5 Fig PrP alters kinesin-1 based anterograde fast axonal transport instantaneous velocity. The effects of Prion on kinesin-1 and dynein based mitochondria fast axonal transport instantaneous velocities were analyzed in 3 days *in vitro* rat embryonic primary hippocampal neurons by time-lapse microscopy. Quantification of the instantaneous velocities of mobile mitochondria was calculated over 3 frames during 10 seconds in the anterograde (red) and retrograde (blue) direction. Mean ±SEM, * p<0.05, total of 143 mitochondria were analyzed; 57 mitochondria were analyzed in scramble treated neurons 26 (Not mobile), 12 (0.963±0.041μm/sec. anterograde direction), 19 (0.747±0.041μm/sec. retrograde direction); 86 mitochondria were analyzed in PrP_106-126_ treated neurons, 58 (not mobile), 7 (0.398±0.070μm/sec. anterograde direction), 21 (0.685±0.160μm/sec. retrograde direction). Results were obtained from 3 independent experiments. One-way ANOVA with post-hoc Tukey.
(TIF)

S1 Table (PDF)
