# Supplementary figures and images for: Prion protein inhibits fast axonal transport through a mechanism involving casein kinase 2
Source: PLoS One. 2017 Dec 20;12(12):e0188340. doi: 10.1371/journal.pone.0188340 (PMC5737884; doi:10.1371/journal.pone.0188340)

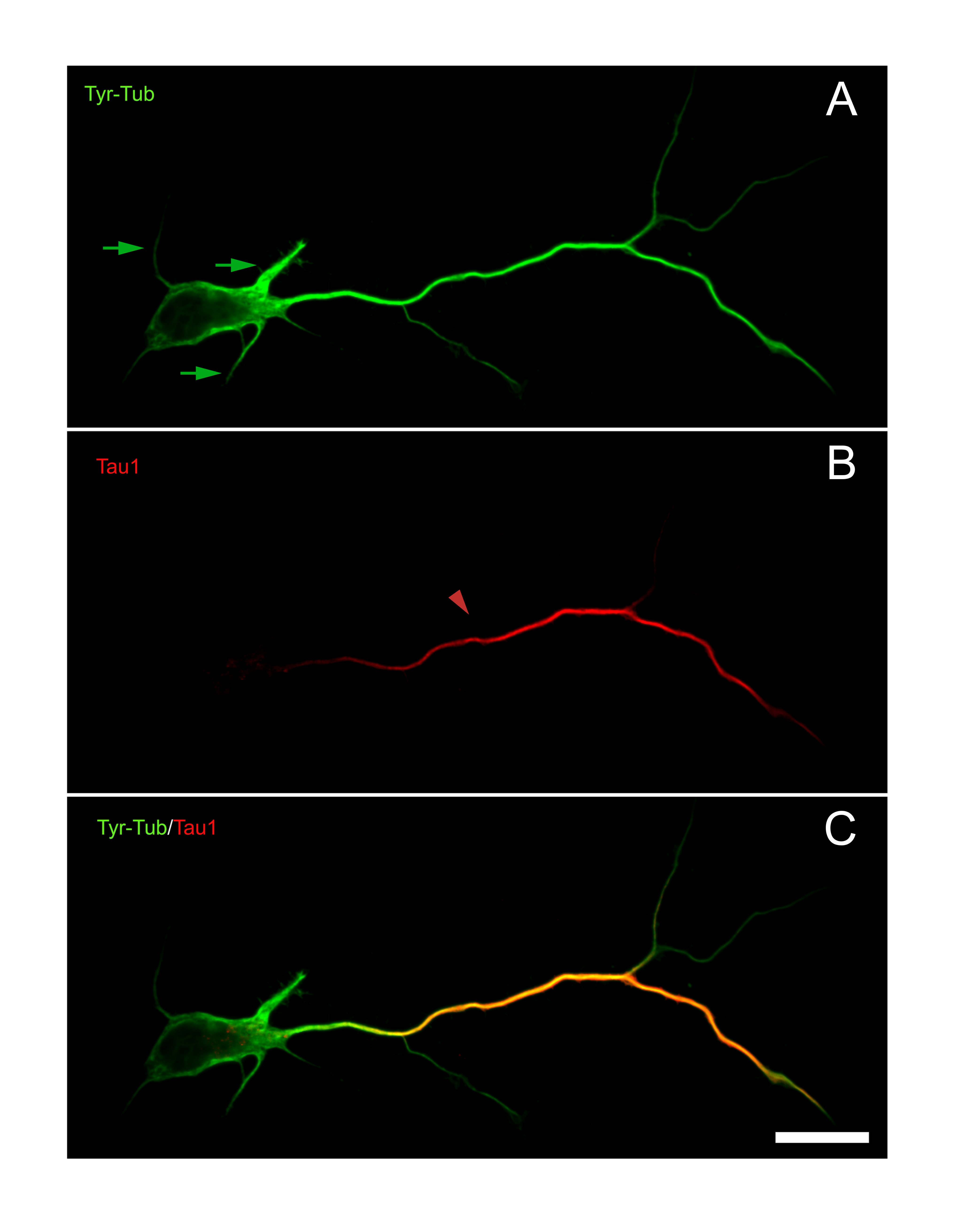

Supplement: S1 Fig — Confocal image showing a 3 days in culture hippocampal neuron immunostained with a monoclonal antibodies against (A) tyrosinated tubulin (tyr-Tub; green and green arrows) and (B) dephosphorylated Tau (Tau-1; red and red arrowhead). (C) superimposition of A and B. Note the axonal distribution of tau along the major process versus the widespread localization of tubular within the minor processes and cell body. Scale bar 20μm. (TIF) [file pone.0188340.s001.tif]

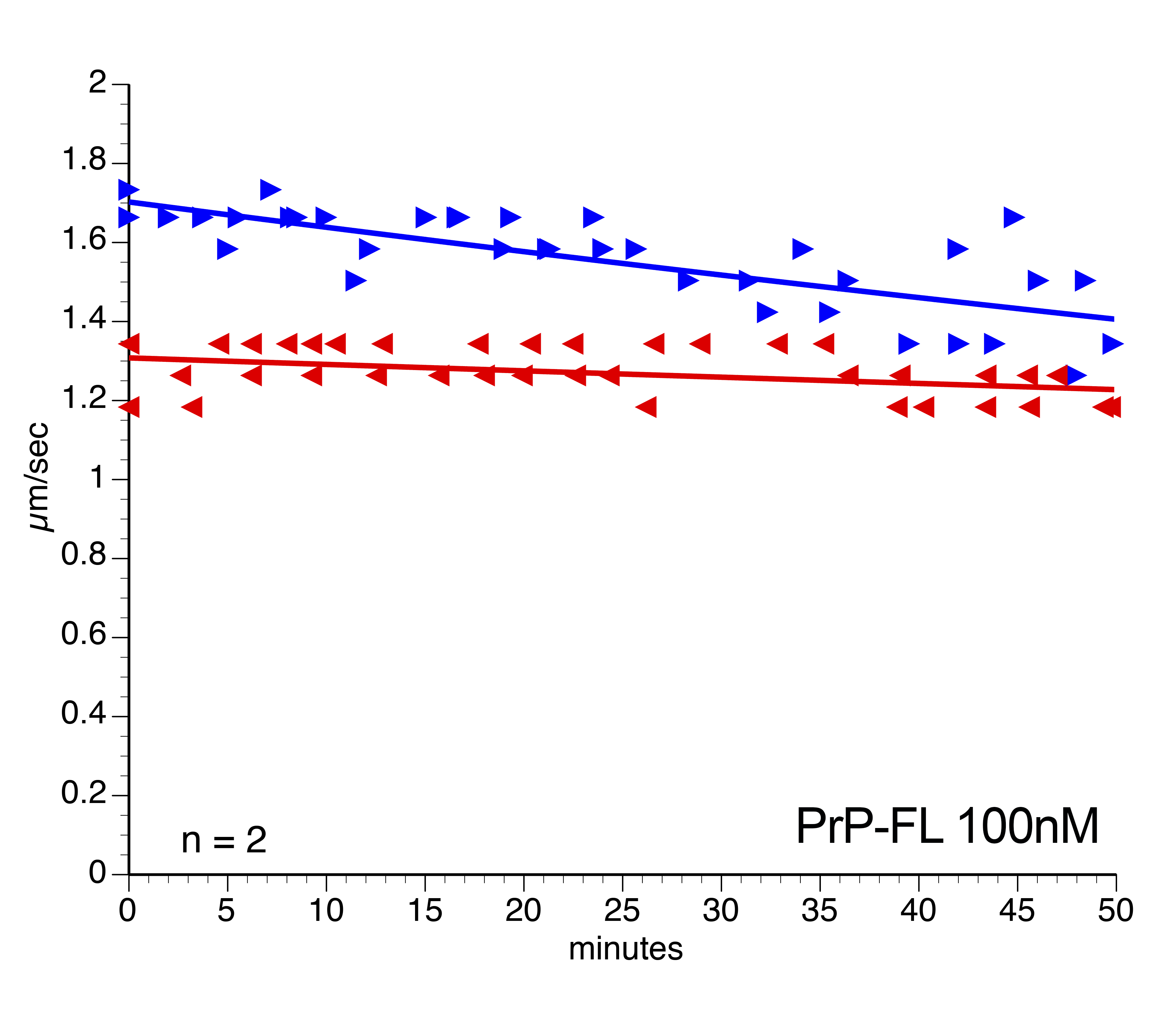

Supplement: S2 Fig — Plot represents results from vesicle motility assays in isolated extruded squid axoplasms perfused with PrP-FL at 100nM concentration. Blue arrowheads and blue line represent fast axonal transport (FAT) rates of kinesin-1 driven vesicles moving in the anterograde direction and the red arrows and red lines represent retrograde dynein-mediated FAT rates. Lines represent the best fit exponential of rates for vesicles moving in the anterograde blue arrows and retrograde red arrows directions over time in axoplasms. Perfusion with 100nM of PrP-FL showed a marked reduction of anterograde and a modest retrograde FAT soon after perfusion, compared to perfusing X/2 buffer alone [48] (data not shown in this manuscript) or PrP-Scram (Fig 1D). (TIF) [file pone.0188340.s002.tif]

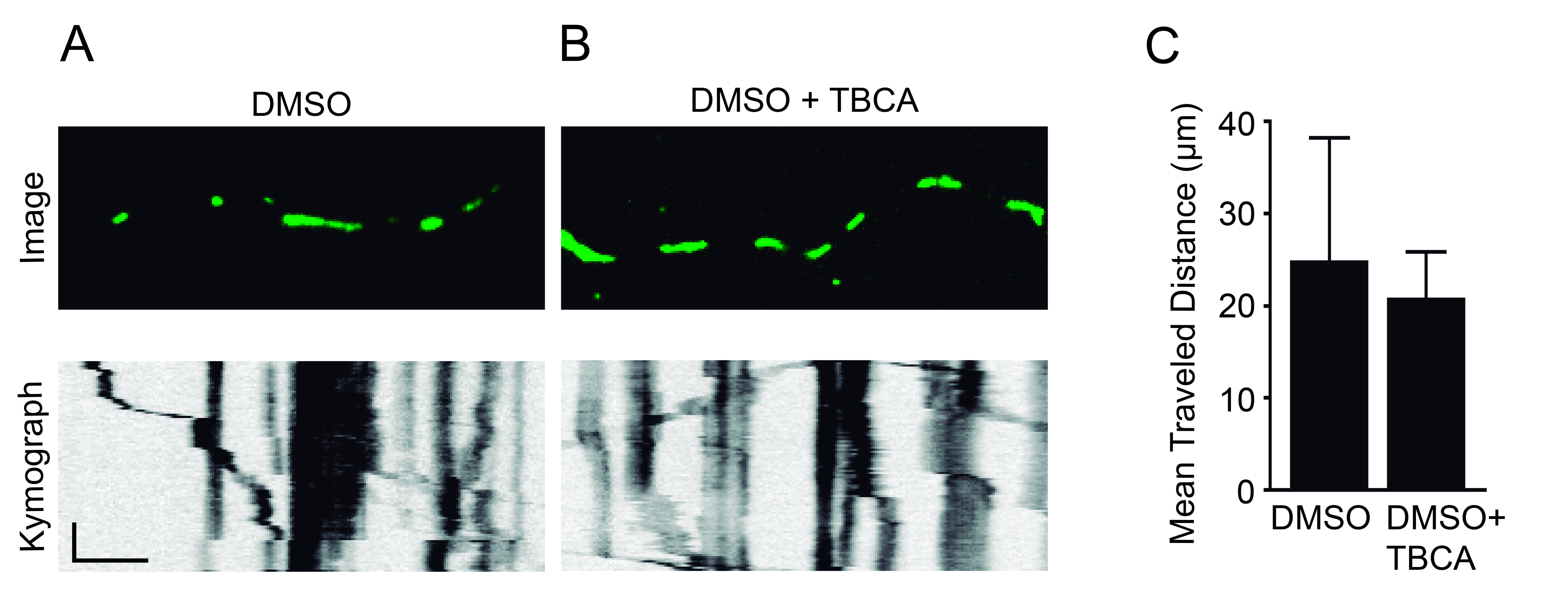

Supplement: S3 Fig — Upper panel shows fluorescently labeled mitochondria from axons of 3 DIV neurons treated with either vehicle (DMSO) or the CK2 inhibitor DMAT 5μM for 60 min. In the lower panel, kymographs reveal the trajectory of mitochondria motility from neurons incubated with vehicle (A) or (B) 5μM DMAT for 1 hour. (C) Quantification of average distance traveled by mitochondria as analyzed in (A) 24.88±13.47 μm and (B) 20.76±5.16 μm in the retrograde direction. Note the lack of effect when neurons are incubated with 5μM DMAT alone compared to DMSO treated neurons. Scale bar in the X-axis equals 30μm and in the Y-axis equals 60 seconds. Mean ±SEM, total of 19 neurons were analyzed, 8 (Control DMSO treated) and 11 (DMSO+DMAT treated). Results were obtained from 3 independent experiments. One-way ANOVA with post-hoc Tukey. (TIF) [file pone.0188340.s003.tif]

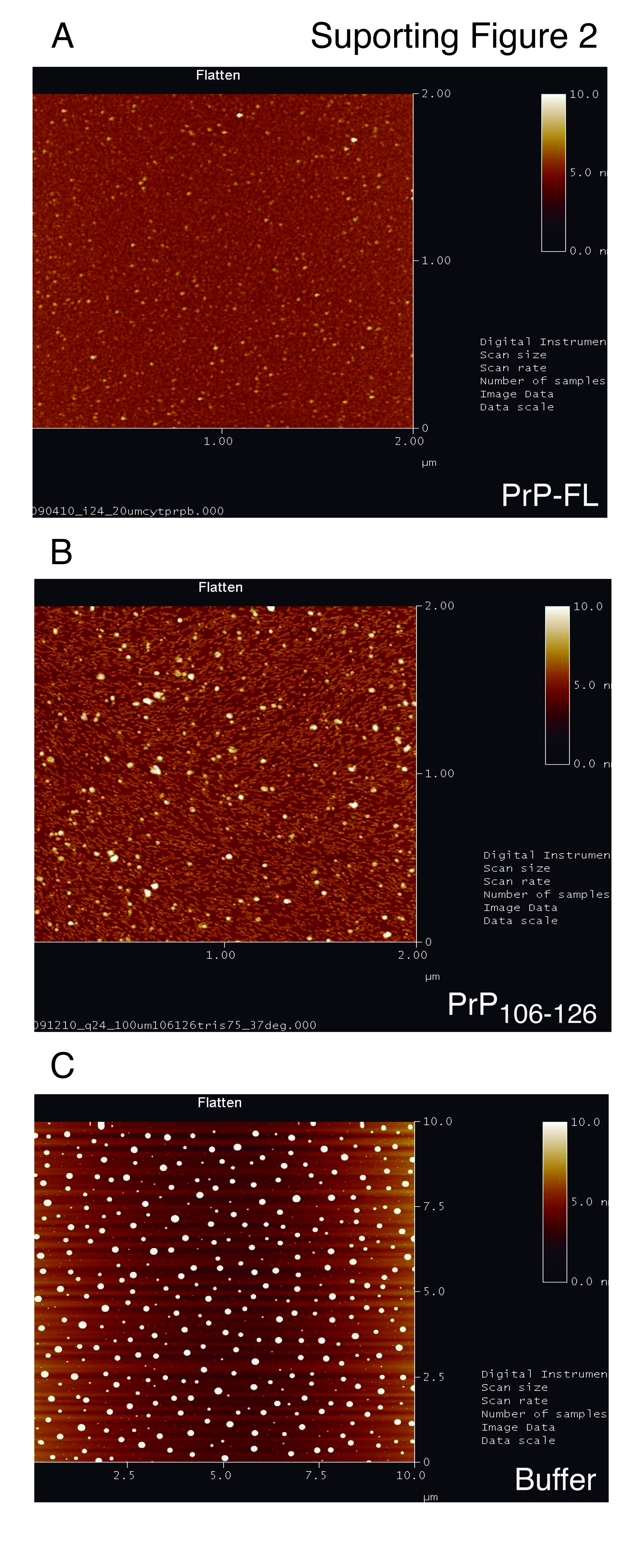

Supplement: S4 Fig — AFM analysis showed a common oligomeric structure present in PrP-FL and PrP106-126. (A) Recombinant PrP-FL (1mM) and (B) synthetic PrP106-126 peptide resuspended in H2O at 1mM concentration were incubated at 37°C for 60 minutes. (C) Buffer x/2 alone. Solutions were diluted to 20μM with the same buffer used for perfusing squid axoplasms (Buffer X/2) and then analyzed on mica under ambient conditions using tapping mode AFM. White round dots in A-C seem to be attributed to salt in the X/2 buffer. All AFM images shown are 2_2-mm x–y, 10nm total z-range. (TIF) [file pone.0188340.s004.tif]

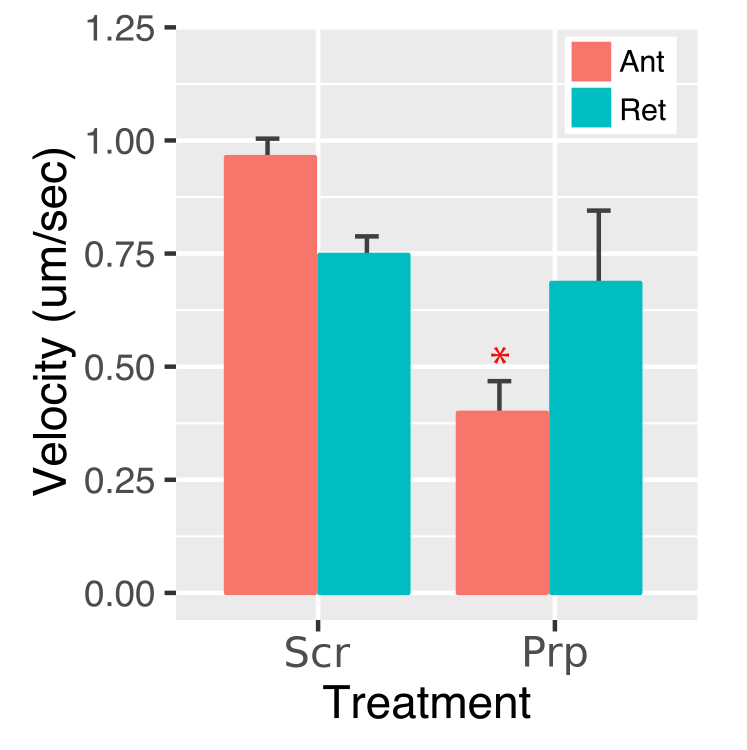

Supplement: S5 Fig — The effects of Prion on kinesin-1 and dynein based mitochondria fast axonal transport instantaneous velocities were analyzed in 3 days in vitro rat embryonic primary hippocampal neurons by time-lapse microscopy. Quantification of the instantaneous velocities of mobile mitochondria was calculated over 3 frames during 10 seconds in the anterograde (red) and retrograde (blue) direction. Mean ±SEM, * p<0.05, total of 143 mitochondria were analyzed; 57 mitochondria were analyzed in scramble treated neurons 26 (Not mobile), 12 (0.963±0.041μm/sec. anterograde direction), 19 (0.747±0.041μm/sec. retrograde direction); 86 mitochondria were analyzed in PrP106-126 treated neurons, 58 (not mobile), 7 (0.398±0.070μm/sec. anterograde direction), 21 (0.685±0.160μm/sec. retrograde direction). Results were obtained from 3 independent experiments. One-way ANOVA with post-hoc Tukey. (TIF) [file pone.0188340.s005.tif]
