# Supplementary material for: Prion protein inhibits fast axonal transport through a mechanism involving casein kinase 2
Source: PLoS One. 2017 Dec 20;12(12):e0188340. doi: 10.1371/journal.pone.0188340 (PMC5737884; doi:10.1371/journal.pone.0188340)
Supplement: S1 Table — (PDF) [file pone.0188340.s006.pdf]

S1 Table 1. The central domain of PrP is necesary and sufficient to inhibit FAT

Anterograde Fast Axonal Transport Rates						
**Experiment**	**n**	**# rate measurements**	**Av. Rate (µm/sec)**	**Variance**	**Std Dev**	**Std Err**
Prion-Scram	3	23	1.657	0.00022	0.01486	0.00858
Prion-FL	4	16	1.275	0.00983	0.09914	0.04957
$Prion_{106\text{-}126}$	4	15	1.122	0.00882	0.09389	0.04695
Prion ΔCD	3	19	1.631	0.01155	0.10749	0.06206
Prion-FL + DMAT	3	21	1.573	0.01198	0.10946	0.06320
$Prion_{106\text{-}126}$ + DMAT	3	23	1.608	0.00610	0.07813	0.04511

Retrograde Fast Axonal Transport Rates						
**Experiment**	**n**	**# rate measurements**	**Av. Rate (µm/sec)**	**Variance**	**Std Dev**	**Std Err**
Prion-Scram	3	22	1.262	0.00350	0.05914	0.03414
Prion-FL	4	16	1.092	0.00645	0.08029	0.04014
$Prion_{106\text{-}126}$	4	15	0.940	0.01217	0.11033	0.05517
Prion ΔCD	3	17	1.210	0.00124	0.03526	0.02036
Prion-FL + DMAT	3	21	1.185	0.00320	0.05658	0.03267
$Prion_{106\text{-}126}$ + DMAT	3	22	1.224	0.00022	0.01475	0.00851

n: Number of independent experiments.
# rate measurements: Number of indepemdemt fast axonal transport rates measurements obtained from 30-50 minutes after perfusion.
Av. Rate: Average fast axonal transport rates
Std Dev: Standad deviation.
Std Err: Standard error.
